# Gender-Specific Response in Pain and Function to Biologic Treatment of Knee Osteoarthritis: A Gender-Bias-Mitigated, Observational, Intention-to-Treat Study at Two Years

**DOI:** 10.1155/2021/6648437

**Published:** 2021-02-25

**Authors:** Tiffanie-Marie Borg, Nima Heidari, Ali Noorani, Mark Slevin, Angela Cullen, Stefano Olgiati, Alberto Zerbi, Alessandro Danovi, Adrian Wilson

**Affiliations:** ^1^Academic Plastic Surgery Group, Barts and the London School of Medicine, UK; ^2^The Regenerative Clinic, 18-22 Queen Anne Street, London, UK; ^3^nextAI, London, UK; ^4^Department of Life Sciences, Manchester Metropolitan University, UK; ^5^Department of Morphology, Surgery and Experimental Medicine, Faculty of Medicine, University of Ferrara, 44121 Ferrara, Italy; ^6^University of Milano, Department of Radiology, 20100 Milano, Italy; ^7^Department of Quantitative Methods, University of Bergamo, 24129 Bergamo, Italy

## Abstract

Knee osteoarthritis is a major cause of disability worldwide. Newer modalities of treatment with less morbidity, such as intra-articular injection of microfragmented fat (MFAT), are showing promise. We report on our novel observation that women show a greater improvement in pain and function to MFAT than men. Traditionally, women have been underrepresented in studies and studies with both sexes regularly fail to analyze the results by sex. To mitigate for this bias and quantify it, we describe a technique using reproducible statistical analysis and replicable results with Open Access statistical software R to calculate the magnitude of this difference. Genetic, hormonal, environmental, and age factors play a role in our observed difference between the sexes. There is a need for further studies to identify the molecular basis for this difference and be able to utilize it to improve outcome for both women and men.

## 1. Introduction

Osteoarthritis (OA) is a major cause of disability [1] and mortality [2] in an aging population. By eighty years of age, there is radiographic evidence of osteoarthritic joint degeneration in almost all individuals [3]. However, life expectancy is projected to exceed this at a global scale; Bayesian forecasting models suggest that by 2030 there is a 50% probability that the female life expectancy will exceed ninety years and a 95% chance that the male life expectancy will exceed eighty years [4]. It is therefore imperative that the management and response to treatment are optimised and account for inherent differences in response to treatment according to age, gender, and ethnicity. Current management includes nonsurgical therapies [5–7] and surgical intervention.

In view of the risks associated with surgery, there has been growing interest in nonsurgical therapies. Microfragmented adipose tissue (MFAT) is one such therapy and is being increasingly explored for its benefit in managing OA. Cell-based therapies have demonstrated potential in treating OA [8, 9]. Mesenchymal stem cells (MSCs), derived from MFAT, can transform into cartilage, adipocytes, osteoblasts, and osteocytes [10]; inhibit T cell growth [11]; and encourage joint repair through cartilage regeneration and inflammatory downregulation. They also relieve OA pain by producing cytokines that target neurogenic pain pathways [12]. The role of microfragmented adipose tissue (MFAT) to optimise the joint environment during surgery is promising; authors of studies relating to MFAT and OA consistently conclude that MFAT is safe [13] and effective, increasing glycosaminoglycan (GAG) cartilage content [14] to result in improved joint function, pain, and quality of life [9, 13, 15–21].

Differences in disease pathology and response to treatment exist between individuals. One reason for these differences is gender. This has been demonstrated even in rates of morbidity and mortality following the coronavirus outbreak [22]. A number of studies have demonstrated a difference in outcome following orthopaedic intervention between genders [23]. Analysis of 698 patients who underwent elective total knee arthroplasty by Parsley et al. [24] revealed that women are more likely to seek treatment at a later stage of joint degeneration than men. Katz et al. [25] similarly reported worse functional status in women prior to total knee arthroplasty (*p* < 0.01), total hip arthroplasty (*p* < 0.01), and laminectomy for spinal stenosis (*p* < 0.01). Women are at higher risk of developing adverse local tissue reactions, dislocation, aseptic loosening, and need for revision surgery following primary metal-on-metal hip resurfacing arthroplasty [26]. However, postoperatively, women report greater improvement in functional scores and quality of life [27].

To our knowledge, the difference in response between men and women has not been reported in biologic treatment for knee OA. We hypothesise that women and men demonstrate similar outcomes following treatment of knee OA with MFAT.

## 2. Materials and Methods

This observational, intention-to-treat study included the complete sample of 456 patients who agreed to be scored for pain (visual analogue scale (VAS)) and function (Oxford knee score (OKS)) at baseline regardless of subsequent changes to adherence or status during follow-up. All patients attended the private clinics of the authors (AW, NH) complaining of knee pain with a diagnosis of knee osteoarthritis.

The study was conducted in accordance with the principles of Good Clinical Practice (NIHR) and the General Medical Council (GMC) guidelines on research, patient consent to research, and future publication, as well as adhering to and in accordance with the Declaration of Helsinki. This study was carried out in a private practice setting.

All patients were clinically reviewed and physically examined by an orthopaedic surgeon. The preoperative assessments included evaluation of imaging (X-ray in all cases and MRI in some) where the knee OA was graded using the Kellgren and Lawrence (KL) grading system [28].

### 2.1. Inclusion Criteria

The inclusion criteria were as follows: no deformity greater than ten degrees of varus or valgus and the presence of knee OA as diagnosed on X-ray and/or MRI.

Exclusion criteria included the following: recent injury (<3 months) of the symptomatic knee, infectious joint disease, malignancy, pregnancy, anticoagulation or thrombocytopenia, coagulation disorder, and intra-articular steroid injections performed within the last three months.

The patients were informed of all possible options for treating their knee OA including conservative means, injections of a number of substances including steroids, hyaluronic acid, platelet-rich plasma, and microfragmented adipose tissue. They also had surgical options detailed to them including osteotomy and partial and total knee replacement.

### 2.2. Statistical Methods

#### 2.2.1. Reproducibility of Analysis and Replicability of Results

In order to make statistical analysis reproducible and results replicable, we utilized Open Access software R version 4.0.3 (2020-10-10) and later. In addition, all figures have been generated automatically by software R and are therefore reproducible and replicable.

#### 2.2.2. Gender Bias by Imbalanced Gender Representation in the Dataset

In the complete sample of 418 patients, women were underrepresented, with an imbalance of 192 females versus 226 males. This imbalance in the dataset can generate a bias in a class-specific analysis, in our case a gender bias in the minority class which is the class representing women [29].

**Figure 1:**
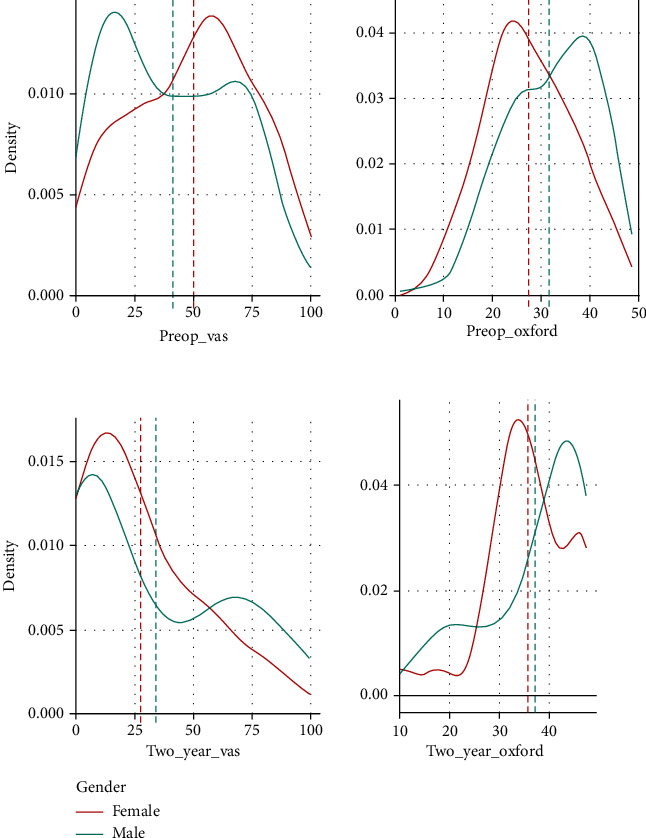
Preoperative and 2-year (postoperative) VAS and OKS density distribution prior to gender bias mitigation. The *x*-axis shows VAS (0-100) and OKS (0-48) pre- and 2 years post MFAT injection. The *y*-axis shows the density distribution of the variables. Source: authors' data and reproducible statistical analysis with Open Access statistical software R (version 4.0.0 or higher).

**Figure 2:**
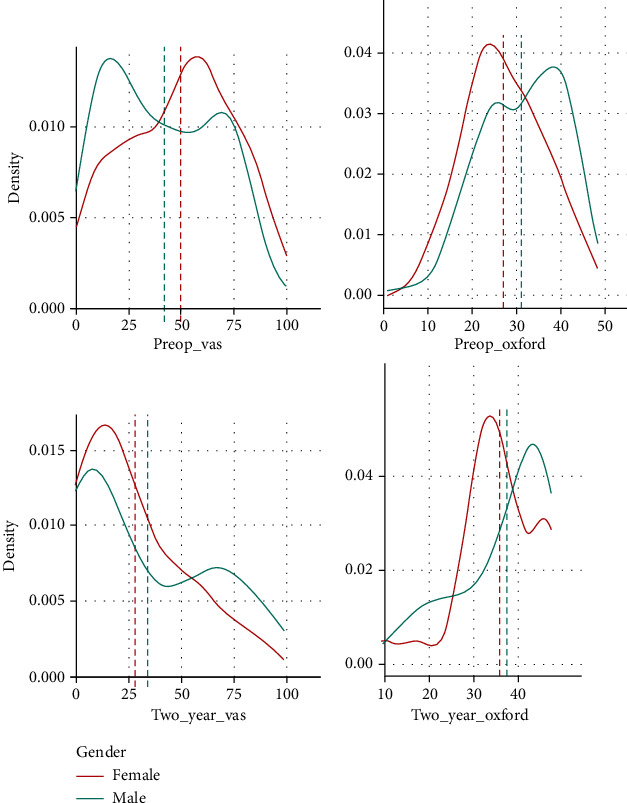
Preoperative and 2-year (postoperative) VAS and OKS density distribution after gender bias mitigation. The *x*-axis shows VAS (0-100) and OKS (0-48) pre- and 2 years post MFAT injection. The *y*-axis shows the density distribution of the variables. Source: authors' data and reproducible statistical analysis with Open Access statistical software R (version 4.0.0 or higher).

To equalize the male and female samples for further analysis, we performed a random undersampling of the cohorts using an algorithm. This produced 192.4 females and 193.6 in the male cohort. Rounding these numbers produced 192 patients in the female and 194 in the male cohorts. The difference of 2 patients cannot be removed as this would need to be done manually, making the decision subjective and nonrandom therefore nonreproducible. The density distribution of the VAS and OKS was plotted prior to (Figure 1) and post (Figure 2) random undersampling of the dataset. These demonstrate the preservation of the class distribution of the data. All further analysis was performed on the gender-bias-mitigated dataset.

#### 2.2.3. Missing Values and Imputation

**Figure 3:**
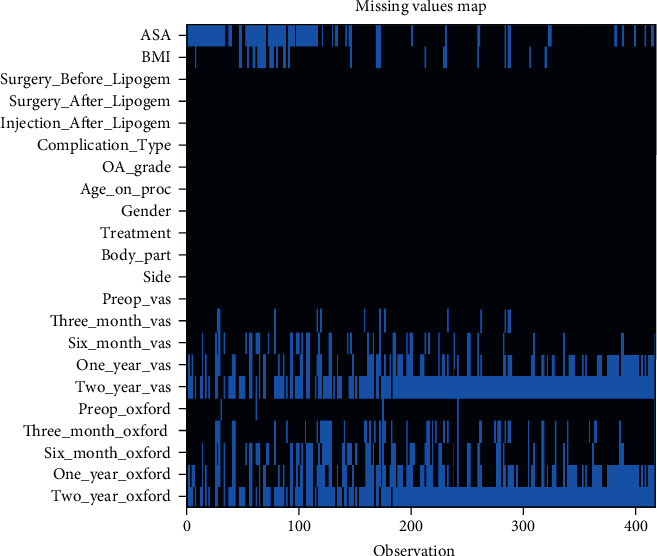
Missingness map. Our dataset consisted of 22 variables for each of the 418 knees. This amounts to a total of 9196 data points. Missing data were missing completely at random (MCAR), with a missingness rate of 17% (light blue) and 83% observed (dark blue). Source: authors' data and reproducible statistical analysis with Open Access statistical software R (version 4.0.0 or higher).

Our dataset consisted of 418 sets of observations and 22 variables per set of observation, for a total of 9196 data points (Figure 3). Missing data were missing completely at random (MCAR), with a missingness rate of 17% (83% observed; 17% missing).

Missing data is a frequent problem in clinical studies, in particular when long-term follow-ups are involved. With longer the follow-ups, patients tend not to adhere to controls and not to answer every question in patient-reported questionnaires. Most statistical analysis methods however “… assume the absence of missing data and are only able to analyze observations for which every variable is measured and reported. Dropping partially observed observations from a statistical analysis is a potential cause of biases, inefficiencies, and incorrect uncertainty estimates” [30].

Another approach is to fill in or rectangularize incomplete datasets so that “analyses which require complete observations can appropriately use all the information present in a dataset with missingness” [31].

Also, missing data imputation is a potential source of bias, but in the imputation approach, information is not lost as it would be in missing data elimination, because imputation algorithms are reproducible, results are replicable, and reliability and bias can be assessed transparently by reviewers and readers.

For these reasons, we utilized R software package Amelia II to impute missing data by multiple imputation. Multiple imputation “involves imputing *m* values for each missing cell in your data matrix and creating *m* ‘completed' datasets. Across these completed datasets, the observed values are the same, but the missing values are filled in with different imputations that reflect our uncertainty about the missing data” [31]. This allows us to avoid the biases, inefficiencies, and incorrect uncertainty estimates that can result from dropping all partially observed observations from the analysis.

After imputation, we applied a Bayesian statistical method to analyze the complete dataset [32].

### 2.3. Statistical Analysis

We performed a Bayesian analysis for the two groups of women and men to provide the reader with a complete representation of the uncertainty underlying our estimates with information about the samples and the credible values for the group means and their differences [33, 34].

For one product approved in the US, Dermagraft®, Bayesian statistics were used to evaluate product efficacy, instead of traditional (frequentist) statistics. Based on the statistical guidance for clinical trials recently issued by the US Food and Drug Administration [35, 36], statistical analyses including Bayesian statistics are key elements in the design of clinical trials for products based on human cells and tissues. New regulations regarding human cells and tissue products have recently been implemented in Japan, including conditional and time-limited approval for regenerative medicine products. In these cases, Bayesian statistics are a promising alternative approach to support product development.

We utilized the R BEST Package developed by Meredith and Kruschke [37]. We believe that the BEST Package provides a Bayesian alternative to a *t*-test, providing much richer information about the samples, the difference in means, and the underlying uncertainty in the estimates than a single *p* value.

Since, to our knowledge, there is no existing study measuring gender-specific response in pain and function to biologic treatment of knee osteoarthritis, we decided to assume that we have no prior knowledge on the comparative responses, and we utilized minimally informative priors: i.e., normal priors with a large standard deviation for the mean and a broad uniform priors for standard deviation, as described by Kruschke [33, 38].

#### 2.3.1. Patients

The mean age at the time of the procedure for women was 66 (SD 12) and 65 (SD 12) for the men (Table 1). Both groups had moderate to severe OA with a median grade of 3 [28]. The mean BMI for women was 28 (SD 5) with a median American Society of Anesthesiology (ASA) [39] grade of 2. The men had similar BMI of 27 (SD 4) and ASA of 2 (Table 2). Further breakdown of the KL grade for OA is provided in Table 1.

Full and informed consent was undertaken for each part of the procedure including sedation, lipoaspiration, and image-guided intra-articular injection. All procedures were performed in an operating theatre as a day case, and patients were discharged approximately three hours following the completion of the procedure.

### 2.4. Harvesting the Adipose Tissue and Injecting MFAT

Adipose tissue was harvested and microfragmented using previously published technique [9]. The MFAT was then injected under ultrasonographic guidance into the knee joint. The procedure was performed under sedation in an operating theatre. Following full recovery, the patients were discharged with a physiotherapy protocol.

Outcomes were measured using the visual analogue scale (VAS) for pain and the Oxford knee score (OKS) for function. All patients completed these questionnaires before treatment and at three months, six months, one year, and 2 years following treatment.

VAS is a validated measurement system that allows participants to measure their pain intensity along a continuous scale of values that otherwise cannot clearly be measured [40]. Participants are presented with a horizontal line that is anchored by two extremes, between 0 and 100 (0 = no pain, 100 = worst pain), and are asked to place a point along the VAS line at the point that would represent their current level of pain.

OKS consists of 12 questions, scored 0-4 with 0 being severe and 4 being no symptoms, covering pain and function of the knee [41]. The best outcome is a score of 48 and the worst a score of 0. We chose the OKS as the best performing condition specific score [42] for patients with severe OA that would have been candidates for arthroplasty.

Adverse events and complications were recorded (Table 3). The most serious was a female patient who required knee washout due to continued pain and inflammation. This was performed at another institution, and further details are not available. The most common issues included joint pain and pain at the fat harvest site. We only noted the joint pain as a complication if it required more analgesia than was prescribed as a part of the standard postoperative pack. This data is from the full dataset to present the full spectrum of adverse events. As these are relatively small numbers, we did not wish to have any removed from reporting by the random undersampling of the dataset.

#### 2.4.1. Responder Classification

Patients were categorized according to whether they have had an improvement (responder) or no improvement (nonresponder) following the injection of MFAT into their knee. We found three groups in each outcome parameter. They have been termed superresponder, responder, and nonresponder. For the VAS, all those who did not show an improvement we termed nonresponder, and those who showed an improvement of 1 to 19 points greater than pretreatment on the scale we termed a responder. Those who had an increase of 20 points or more were termed a superresponder [43].

For the OKS, all those who did not show an improvement we termed nonresponder, and those who showed an improvement of 1 to 6 points greater than pretreatment on the scale we termed a responder. Those who had an increase of 7 points or more were termed a superresponder [44].

Both the superresponder categories reflect the level of improvement in these outcome measures that have been deemed to be the minimum clinically important difference (MCID).

## 3. Results

### 3.1. General Outcomes

The results are reported following mitigation of gender bias and analysis of the balanced dataset. Mean preoperative VAS (prior to the injection of MFAT) was 49 in women and 41 in men. The mean VAS at 2 years after MFAT injection was 28 for women and 34 for men (Table 4). The full distribution density of these is displayed graphically in Figure 2.

The mean OKS preinjection was 27 for women and 32 for men. The mean OKS 2 years after the injection was 36 for women and 38 for men (Table 4). The full distribution density of these is displayed graphically in Figure 2.

**Figure 4:**
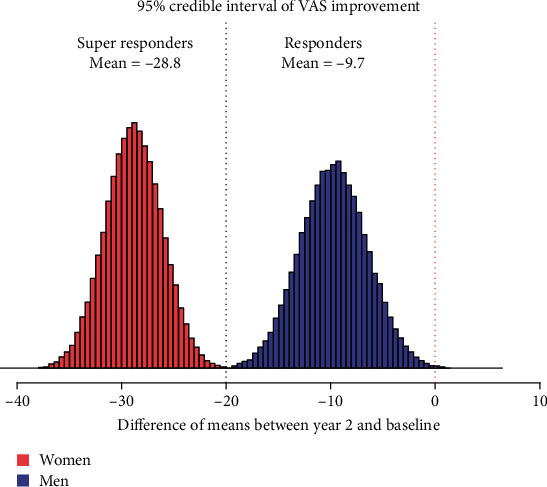
Gender-specific mean VAS score improvement at year 2 versus baseline. Source: authors' data and reproducible statistical analysis with Open Access statistical software R (version 4.0.0 or higher).

The difference of means between the pre- and postinjection VAS and OKS is tabulated in Table 5 and demonstrated a credible reduction of -28.8 (95% CI: -23.6–-34.4) in VAS for women and a reduction of -9.7 (95% CI: -3.0–-16.0) for men.  Figure 4 shows the Bayesian plot and the entire uncertainty distribution of the credible reduction in pain for male and female patients with the responder and superresponder thresholds marked.

**Figure 5:**
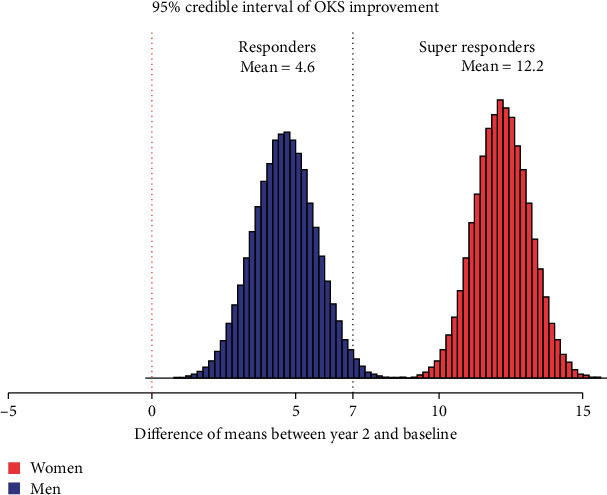
Gender-specific mean OKS score improvement at year 2 versus baseline. Source: authors' data and reproducible statistical analysis with Open Access statistical software R (version 4.0.0 or higher).

The same pattern is seen with the OKS where the women have a credible improvement of +12.2 (95% CI: +10.3–+14.1) and the men +4.6 (95% CI: +2.5–+6.8) (Table 5). The data presents a greater and more credible improvement both in pain and function for women.  Figure 5 shows the Bayesian plot and the entire uncertainty distribution of the credible improvement in function (OKS) for male and female patients with the superresponder threshold marked.

### 3.2. Response to Treatment

#### 3.2.1. VAS

In the female group, a total of 164 of 192 (90%) responded to the treatment, with 123 (64%) being superresponders seeing a 20 or more drop in their VAS score for pain (Table 6).

In the male group, 117 of 194 (60%) responded by showing an improvement to the treatment, with 78 (40%) being superresponders seeing a 20 or more drop in their VAS score for pain (Table 6).

#### 3.2.2. OKS

In the female group, a total of 167 of 192 (87%) responded to the treatment, with 134 (70%) being superresponders seeing an improvement of 7 or more in the OKS functional score (Table 6).

In the male group, 126 of 194 (65%) improved with the treatment, with 74 (38%) being superresponders seeing an improvement of 7 or more in the OKS functional score (Table 6).

## 4. Discussion

Our study highlights a difference in outcomes between men and women following MFAT treatment of knee OA. We report that a greater proportion of women responded to treatment than men: 90% vs. 60% change in VAS scores with 87% vs. 65% change in OKS scores for women and men, respectively. Of those who responded to MFAT, women demonstrated the greater improvement in pain and function. Preinjection, women were in more pain with worse joint function. However, two years after MFAT treatment, they demonstrated a greater reduction in discomfort with superior joint function.

Though this is the first report of different outcomes between the sexes following MFAT therapy, there is increasing supporting evidence that men and women with similar pathologies respond differently to both medical and surgical treatments [45]. Basques et al. reported the outcomes of 6,123,637 patients who underwent a total hip or knee arthroplasty [46]. Men had a statistically significant higher rate of acute kidney injury, wound dehiscence, surgical site infection, sepsis, pneumonia, myocardial infarction, cardiac arrest, and death (*p* < 0.001) while women had higher rates of urinary tract infections, deep vein thrombosis, and requirements for blood transfusion (*p* < 0.001). These results are corroborated by a multivariate analysis of gender and postarthroplasty complications performed by Robinson et al. [47]. Likewise, the authors concluded that posttotal hip/knee arthroplasty, complications vary between men and women with gender serving as an independent risk factor for certain complications.

There is also a gender difference in the clinical presentation and treatment outcomes between men and women managed by other medical specialties. Response to cancer immunotherapies [48] and transplantation [49] and outcomes for cardiovascular disease [50], ischemic stroke [51], traumatic brain injury [52], and movement disorders [53] differ between men and women. Interestingly, gender even plays a role in determining outcome before birth. Male gender independently increases the risk of pregnancy-associated complications [54] including perinatal mortality [55], low Apgar scores [55], umbilical cord problems [56, 57], labour dystocia [58, 59], and foetal distress [55, 60, 61].

The difference in response to MFAT between men and women is likely to be due to underlying genomic, hormonal, and metabolic factors. It is widely known that women experience greater functional impairment with OA than men. A study comparison of physical joint function and insulin-like growth factor revealed growth hormone deficiency in 21% of women and 4% of men undergoing knee arthroplasty [62]. Raised levels of serum leptin [63], parathyroid hormone [64], and oestrogen (both endogenous and through contraceptive/hormone replacement therapy) [65, 66] have also been implicated as risk factors for knee OA. Though existing literature explores the increased pain and worse joint function experienced by women pretreatment, further research is needed to fully understand the difference in response to MFAT between men and women.

Despite the differences between genders, there remains a failure to incorporate sex differences in study designs and analyses. This stems from a long-standing lack of (1) recruitment of women into clinical trials [67], (2) reporting of data pertaining to gender [68], and (3) funding of studies involving the female population [69]. The American National Institutes of Health (NIH) advised the inclusion of gender analysis in clinical trials in 1994 [70]. However, until this policy was introduced, women were entirely prohibited from participating in such studies [69]. This has resulted in advancements in medicine, production of management guidelines, and the release of medications that are used to treat both genders, despite being only based on the analysis of men. Since the introduction of the gender inclusion guideline by the NIH, women in America have been able to enter clinical trials. However, female enrollment has not improved dramatically [71], and gender-related data continues to not be assessed and reported [68]. Failure to report gender-related findings may lead to the false conclusion that there is no difference or that the difference is insignificant. Indeed, only 3% of grants awarded annually by the NIH between 2000 and 2003 were for research on differences between sexes [72].

### 4.1. Study Limitations

For this study, we utilized the complete dataset of 418 patients. However, as shown in the missingness map (Figure 3), 17% of data is missing. Missing data occurred as patients did not always return their outcomes in full. The Amelia II package was used to fill in the missing data in a reproducible, replicable, and robust manner [73]. This method has now been adopted widely and approved by the US FDA and EU EMA [74, 75]. Though we used well-established methods to mitigate bias resulting from missing data, this is technically a source of bias and a limitation to this study. Other sources of bias that have not been quantified in this study include ethnicity, age, BMI, and the grade of arthritis. These will be the subject of future analyses, but the extent to which they play a part has not been addressed here.

The use of MFAT is slowly becoming accepted as treatment for the pain of KOA, and a number of publications over the past few years are testament to this. However, one of the issues with our study is the lack of a control group.

The underlying genomics, hormonal profiles, and serum markers involved in the gender difference highlighted by this study have not been taken into account here and require further investigation.

## 5. Conclusions

Identification of the difference between men and women in the response to biologics is new and has not been previously reported. We believe that future studies should use similar protocols to identify gender bias by balancing the dataset correctly to be able to measure the magnitude of the difference between the genders.

We approached this issue by addressing the missing values within our dataset by imputation. The datasets for male and female patients were then balanced. We then used Bayesian methodology to calculate the magnitude of the difference between the genders. All of the analysis was carried out by reproducible statistical analysis with Open Access statistical software R (version 4.0.0 or higher). This way, bias within the collected data is mitigated in order to allow the magnitude of the difference in response between the genders to be demonstrated and quantified. Our results emphasize the benefit of considering cogitating statistical methods, such as Bayesian statistics, when designing clinical trials for regulatory purposes [35, 36].

Further research is required to explore the reasons for the difference seen in outcomes following MFAT injections for treatment of knee OA between men and women.

## Figures and Tables

**Table 1:** Patients' OA grade before and after gender bias mitigation by randomized undersampling.

Gender	OA grade	Before	After
Female	Total	192	192
	1	6	6
	2	40	40
	3	42	42
	4	104	104

Male	Total	226	194
	1	11	7
	2	38	33
	3	56	50
	4	121	104

Source: authors' data and reproducible statistical analysis with Open Access statistical software R (version 4.0.0 or higher).

**Table 2:** Patients' characteristics before and after gender bias mitigation by randomized undersampling.

Gender	Sample size before/after gender bias mitigation		Mean age on procedure (SD)		Median OA grade		Mean BMI (SD)		Median ASA	
	Before	After	Before	After	Before	After	Before	After	Before	After
Female	192	192	66 (12)	66 (12)	3	3	28 (5)	28 (5)	2	2
Male	226	194	65 (12)	65 (12)	3	3	27 (4)	27 (4)	2	2

Source: authors' data and reproducible statistical analysis with Open Access statistical software R (version 4.0.0 or higher).

**Table 3:** Postprocedure complications (before gender bias mitigation).

Complication	Female	%	Male	%
Joint swelling or pain	19	9	22	9
Harvest site bleeding	2	1	3	1
Pain at harvest site	7	3	9	4
Joint washout	1	0.5	0	0

**Table 4:** The mean VAS and OKS at preoperative and 2-year postoperative time points, prior to and post gender bias mitigation. The full density distributions are displayed in Figures 1 and 2.

Gender	Preoperative VAS		2-year VAS		Preoperative OKS		2-year OKS	
	Before	After	Before	After	Before	After	Before	After
Female	49	49	28	28	27	27	36	36
Male	41	41	34	31	32	32	37	38

Source: authors' data and reproducible statistical analysis with Open Access statistical software R (version 4.0.0 or higher).

**Table 5:** Gender-specific improvement at two-year follow-up after gender bias mitigation by randomized undersampling.

Measure	Outcome	Gender	Difference of the means	95% credible interval of the difference of the means
VAS	Pain reduction (−)	Women	-28.8	-34.4 to -23.6
		Men	-9.7	-16.0 to -3.0

OKS	Function improvement (+)	Women	+12.2	+10.3 to +14.1
		Men	+4.6	+2.5 to +6.8

Source: authors' data and reproducible statistical analysis with Open Access statistical software R (version 4.0.0 or higher).

**Table 6:** Rates of response and nonresponse following MFAT injection to the knee after gender bias mitigation.

Gender		Superresponder	Responder	Total responder	Total nonresponder	Total
Female	VAS	123	41	164	28	192
	OKS	134	33	167	25	192

Male	VAS	78	39	117	77	194
	OKS	74	52	126	68	194

Source: authors' data and reproducible statistical analysis with Open Access statistical software R (version 4.0.0 or higher).

## Data Availability

Data are privately held. The authors are happy to consider requests from the editors.

## Abbreviations

AI: Artificial intelligence
ASA: American Society of Anesthesiology
BMI: Body mass index
FDA: Food and Drug Administration
GMC: General Medical Council
KL: Kellgren and Lawrence
KOA: Knee osteoarthritis
MCAR: Missing completely at random
MFAT: Microfragmented adipose tissue
MSCs: Mesenchymal stem cells
NIH: National Institutes of Health
NSAID: Nonsteroidal anti-inflammatory drug
OA: Osteoarthritis
OKS: Oxford knee score
VAS: Visual analogue scale.
